# Identification and Management of Binge‐Eating Disorder and Bulimia Nervosa in Primary Care Settings: A Qualitative Systematic Review of Healthcare Professionals' and Patients' Perceptions

**DOI:** 10.1002/eat.24568

**Published:** 2025-10-14

**Authors:** Stella Kozmér, Christopher O'Rouke, Natalia S. Lawrence, Jane R. Smith, Samantha B. van Beurden

**Affiliations:** ^1^ University of Exeter Medical School, University of Exeter Exeter UK; ^2^ Dorset Healthcare NHS University Foundation Trust Bournemouth UK; ^3^ Department of Psychology University of Exeter Exeter UK

**Keywords:** binge‐eating disorder, bulimia nervosa, identification, management, primary care, qualitative, systematic review

## Abstract

**Objective:**

To understand how the identification and management of binge‐eating disorder (BED) and bulimia nervosa (BN) in primary care settings are perceived by both patients and healthcare professionals, and how rates of identification and referral for support may be improved.

**Methods:**

A systematic review of qualitative studies was conducted. Eight databases were searched in August 2025. Studies were excluded if they focused on secondary or tertiary care, patients under the age of 16, or anorexia nervosa. Data on sample, type of eating disorder, year of publication, and country were extracted, and thematic synthesis was used to synthesize the data. The quality of studies was assessed using the Critical Appraisal Skills Programme checklist for qualitative research.

**Results:**

Twenty‐five studies were included. Four articles were of high quality; the rest (*N* = 21) were medium with a moderate risk of bias. Most of the studies were from the United Kingdom (*N* = 9), with 14 focusing on healthcare professionals' and 13 on patients' perspectives. The main factors influencing the identification and management of BED/BN were identified to be knowledge and understanding of the conditions, healthcare professional–patient interactions, attitudes and emotions toward BED/BN and the healthcare system, and the existence and accessibility of treatment and referral pathways.

**Discussion:**

The interaction of these factors is discussed in relation to the literature, and gaps in research and clinical practice are identified, such as a limited separation of eating disorder categories, which could help inform the development of strategies to improve the identification and management of BED and BN.

**Summary**

- Many healthcare professionals in primary care do not feel confident identifying or managing binge‐eating disorder and bulimia nervosa, partly due to limited knowledge, unclear guidance, and poor access to specialist services.
- Patients often feel dismissed or misunderstood when seeking help for these eating disorders, which can prevent them from getting the care they need and discourage them from continuing to seek support.
- Improving how these conditions are recognized and treated in primary care will require better training for healthcare professionals, clearer referral pathways, and more inclusive, accessible support services for patients.

## 1 Introduction

Eating disorders (EDs) that involve binge eating, such as binge‐eating disorder (BED) and bulimia nervosa (BN), are among the top three most common EDs globally, affecting approximately 4.2% of the population (Galmiche et al. 2019), with suggestions of a higher true prevalence (Hay et al. 2023). Both BED and BN represent significant public health concerns with profound implications for both physical and mental health outcomes (Giel et al. 2022; Milano et al. 2018). If BED and BN are not identified and managed at an early stage, they can lead to a deterioration in both mental and physical health, including the development of comorbidities or worsening of pre‐ or co‐existing conditions (Waller et al. 2009). These comorbidities can include cardio‐metabolic conditions (Giel et al. 2022; Wassenaar et al. 2019; Abiri et al. 2022; Tith et al. 2020), gastrointestinal issues (Milano et al. 2018; Baenas et al. 2024), and anxiety and depression (Giel et al. 2022; Baenas et al. 2024). Such potential complications would inevitably result in increased demand for further treatment, worsening the quality of life (Sheehan and Herman 2015), and increasing the cost to the healthcare system and wider economy (Tannous et al. 2022; Lee et al. 2021; Streatfeild et al. 2021). Overall, BED and BN are estimated to result in a £3.47bn–£4.6bn annual cost to UK society, including wider costs, such as reduced productivity and social care (Jenkins 2022; PriceWaterhouseCoopers LLP 2015), with similar costs seen across other countries (Lee et al. 2021; Streatfeild et al. 2021).

The role of primary care healthcare professionals (HCPs), such as general practitioners (GPs), in identifying and managing BED and BN is pivotal (Johns et al. 2019; Lebow et al. 2021; Rowe 2020), given that primary care settings act as the first point of contact for individuals with health concerns (NHS England 2024a). Early intervention can improve recovery rates by up to 61% in the case of BN and can mitigate the development of comorbidities, making primary care a critical point for BED and BN identification and management (van Son et al. 2010). Recovery rates for BED have not yet been reported, which is a gap in the literature. However, current evidence suggests significant underdiagnosis and undertreatment of BED and BN in primary care settings globally (Johns et al. 2019; Flahavan 2006). This underdiagnosis could be attributed to multiple factors, including limited consultation time (Lebow et al. 2021; Porter et al. 2023), lack of specialized training among HCPs (Johns et al. 2019; Ayton and Ibrahim 2018; Mahr et al. 2015), and patients' tendencies to conceal their ED behaviors due to stigma or lack of awareness (Lazare et al. 2021; Radunz and Wade 2023). Consequently, many patients may not receive timely or appropriate referrals to specialized care, impeding their access to effective treatment (Lazare et al. 2021). Additionally, studies indicate common negative attitudes among HCPs that can have an adverse impact on identifying and managing BED and BN. For example, Johns et al. (2019) suggest in their systematic review of ED healthcare services that HCPs often demonstrate resentment or disgust toward EDs, resulting in a reluctance to screen for them and a negative experience of primary care for patients (Johns et al. 2019; Lazare et al. 2021). Hence, understanding HCPs' and patients' perceptions related to identifying and managing BED and BN in a primary care setting is essential for improving identification rates, clinical outcomes, the quality of care provided, and consequently, individuals' quality of life.

While the literature offers some insight into the perceptions of patients and HCPs, to the authors' knowledge, no existing reviews clearly differentiate between different EDs in their data analysis. Furthermore, to the authors' knowledge, no review has attempted to synthesize evidence on both HCPs' and patients' perspectives to explore any commonalities and differences in their views. Therefore, this systematic review aims to explore and synthesize the perceptions of HCPs (Galmiche et al. 2019) and patients (Hay et al. 2023) on identifying and managing BED and BN in primary care and to synthesize information on the barriers and facilitators they identify. Additionally, it aims to generate evidence‐based recommendations for improving the identification of BED and BN and timely referral to appropriate treatment.

## 2 Methods

### 2.1 Protocol

The protocol for this review was prospectively registered with the PROSPERO database of systematic reviews (Registration Number: CRD42023394919). This systematic review is reported in accordance with the Preferred Reporting Items for Systematic Reviews and Meta‐Analyses (PRISMA) statement (see Supplementary File 1).

### 2.2 Inclusion and Exclusion Criteria

Original qualitative research in peer‐reviewed sources that explored the perceptions of HCPs and/or patients in relation to the identification and management of BED and/or BN was included in this review. No limitations were set for language, year of publishing, or country of origin. Participants in these studies had to be HCPs working within primary care settings and/or patients with BED and/or BN aged 16 and above, with no further restrictions. Studies on both subthreshold and full‐threshold disorders were included. Primary care HCPs were defined by merging definitions of primary care from the United Kingdom (UK) and the United States (US) and included GPs or family physicians, community pharmacists, dentists, optometrists (NHS England 2024a), obstetrician–gynecologists (Mazzoni et al. 2017), psychological well‐being practitioners (PWPs) (NHS England 2024b) and other allied health professionals based in primary care, such as first point of contact physiotherapists. Studies discussing EDs as an overarching topic were included if BED and BN were a focus in their sample, but excluded if they focused solely on AN. Studies focusing on perceptions about interventions or strategies used for identifying and managing BED/BN were included. Studies without relevant qualitative findings or conducted in secondary and tertiary care were excluded.

### 2.3 Data Sources and Search Strategy

The following five electronic databases were searched in August 2025: MEDLINE, APA PsycINFO, Global Health and EMBASE via OVID, ProQuest Dissertation & Thesis Global, Google Scholar, Web of Science Core Collection, and CINAHL Ultimate via EBSCO. Keyword search terms adapted for each database included, for example: “binge*” or “bulimi*,” “general practi*” or “primary healthcare,” and “view*” or “experience*” (see full search strategy in Supplementary File 2). Reference lists of existing relevant reviews were also searched.

### 2.4 Study Selection

Screening of eligible studies was conducted in two phases. In the first phase, two independent reviewers (S.K. and C.O.) reviewed all titles and abstracts of identified records. A third independent reviewer (S.B.v.B.) reviewed 5% of all records. In the second phase, potentially relevant articles were retrieved in full, and all were assessed independently by two reviewers (S.K. and C.O.) against the inclusion criteria. When reviewing articles that had a general focus on EDs or had a mixed patient sample, references were reviewed carefully with relevance toward BED and BN. If the sample consisted primarily of individuals with AN with no specifications to BN or BED, articles were excluded. For HCP articles, relevancy was determined through discussion with a PWP working in primary care, C.O., who provided clinical insight on whether it was possible to identify and isolate findings specific to the management and assessment of BED and BN. If it was not possible, articles were excluded from data analysis. A third independent reviewer (S.B.v.B.) reviewed 25% of the full‐text records. Disagreements were resolved via discussion.

### 2.5 Data Extraction

Data were extracted (S.K.) on study‐level characteristics such as aims and objectives, design, location, sample characteristics, methods for data collection and analysis, and study findings, as well as authors' interpretations. In articles that reported on HCPs' views on EDs, C.O. provided clinical insight in extracting data relevant to findings specific to BED/BN.

### 2.6 Quality Assessment

Risk of bias was assessed using the Critical Appraisal Skills Programme (CASP) tool for qualitative studies (Critical Appraisal Skills Programme n.d.). A 3‐point rating system (i.e., 1 = not mentioned or poorly justified, 2 = little elaboration provided, or 3 = well justified) for each of the items was adopted (Denford et al. 2020; Moolchaem et al. 2015). In this review, the overall CASP ratings of the included studies were used to describe the quality of the studies for contextual purposes. No exclusions were made based on CASP scores.

### 2.7 Analysis

Thematic methods were used to synthesize the data from included studies following the three steps of thematic synthesis outlined by Thomas and Harden (2008). Studies sampling HCPs and patients were analyzed separately. First, initial codes were created by the first author (S.K.) to describe or summarize relevant text, focusing on the study authors' interpretation. These initial codes were reviewed by a senior independent reviewer (S.B.v.B.) and were further developed in discussion with the first author. In the second stage, codes were organized into descriptive themes. The data in the themes captured and described different perspectives of HCPs and patients. Descriptive themes were further reviewed by a senior reviewer (S.B.v.B.) and discussed. After this stage, the two perspectives were integrated, incorporating input from patient and public involvement (PPI) representatives, as discussed in the following section. The split at the early stages of data analysis ensured that when integrating both perspectives, no data were lost, and differences and similarities between the perspectives could be clearly highlighted. Data on findings and interpretations were extracted from Sections 3 and 4 of the manuscripts. In the third stage, analytical themes were generated based on insights from PPI and interest holders, as well as the descriptive themes. A conceptual model was then created to illustrate the relationship between these themes. These were reviewed by the research team and then further discussed and finalized.

### 2.8 PPI Involvement

A PPI group, consisting of individuals with BED or BN, was established to inform the data analysis and interpretation. An interest holder group consisting of three GPs was also involved in this review. Both groups were involved with parts of the data analysis and supported the research team in interpreting results and ensuring that the perspectives of patients and HCPs were equally represented and appropriately integrated. This was executed by giving a sample of direct quotes from primary sources to the groups to be analyzed and for them to assign proposed descriptive themes if deemed appropriate. Then, the data and descriptive themes were discussed in relation to the PPI representatives' and interest holders' experience and the research question. The team met with each group separately once. Each group focused on the perspective relevant to them; however, the PPI group also engaged with the HCP perspective to support the integration of perspectives. Based on discussions with and suggestions from PPI representatives and interest holders, the team was guided to integrate the two perspectives into a single narrative.

### 2.9 Reflexivity

Our research team comprised a female doctoral academic (S.K.) and senior academics (S.B.v.B., J.S., and N.L.). S.K.'s lived experience influenced the design of the review and was counterbalanced by the non‐lived experience perspectives of the supervisory team. During data selection, S.K.'s lived and research experience in this area was balanced with C.O.'s clinical experience. C.O. works as a PWP and clinical supervisor and is registered in this capacity with the British Association for Behavioral and Cognitive Psychotherapies. PWPs work within NHS Talking Therapies (formerly known as Improving Access to Psychological Therapies) in assessing patients to identify appropriate treatment plans following the NICE Guidelines, and providing low‐intensity cognitive behavioral therapy (CBT) interventions. Where patients would not benefit from low‐intensity CBT, the NHS Talking Therapies service also offers high‐intensity CBT and other modalities, such as counseling and eye movement desensitization and reprocessing. These services operate using a hub‐and‐spoke model, but are traditionally viewed as part of primary care, as patients can self‐refer. While NHS Talking Therapies services are only designed to treat depression and anxiety disorders, PWPs assess a wider range of patient presentations, including BED and BN, either to identify comorbid anxiety or depression or to make appropriate referrals to secondary care for the patient. During data analysis, S.K.'s lived experience may have influenced some descriptions of themes and interpretations. This was discussed with S.B.v.B., who counterbalanced S.K.'s interpretation to ensure it is based on the reported data and bias is reduced.

## 3 Results

### 3.1 Study Selection

**FIGURE 1:**
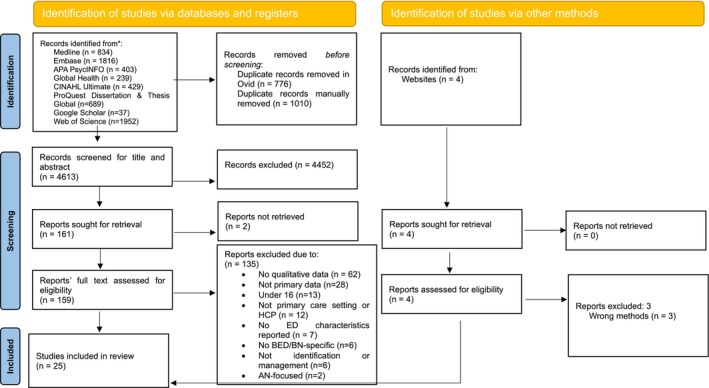
Preferred Reporting Items for Systematic Reviews and Meta‐analyses (PRISMA) flow diagram.

The search results identified 4613 records. Following Title and Abstract screening using our inclusion and exclusion criteria, 4452 were excluded, and 161 full texts were retrieved. Of these, 135 were deemed ineligible for inclusion. After including one additional article retrieved from manual searching, 25 studies were included in the thematic synthesis (see Figure 1). For a list of excluded studies in the full‐text stage (see Supplementary File 4).

### 3.2 Study Characteristics

**FIGURE 2:**
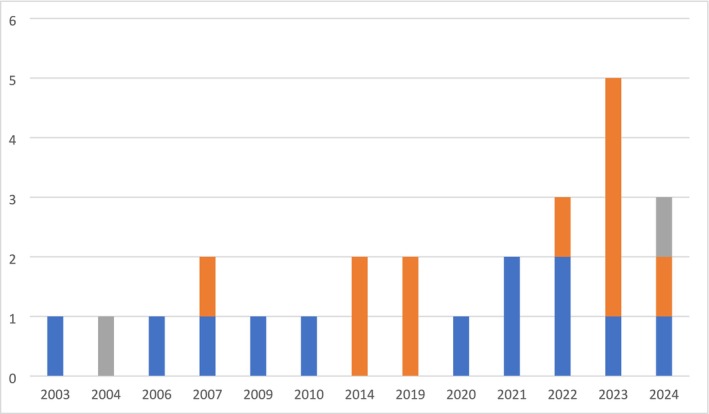
Publishing frequency between 2003 and 2024 (NB: *Y*‐axis—number of articles published, *X*‐axis—year of publication; Blue—HCP perspective, Orange—Lived experience perspective, Gray—Both).

The included studies were published between 2003 and 2024, with the majority published since 2021 (see Figure 2). Nine studies were conducted in the UK (Henderson et al. 2003; Johnston et al. 2007; Malson et al. 2022; Reid et al. 2009; Channa et al. 2019; Räisänen and Hunt 2014; Kinnaird et al. 2019; Ashby and Ogden 2024; Durand 2004), five in the USA (DeBate and Tedesco 2006; Linville et al. 2010; Herman et al. 2014; Ritholz et al. 2022; Masheb et al. 2024), four in Australia (Wade et al. 2022; Banasiak et al. 2007; Patterson‐Norrie et al. 2023; Kwok et al. 2023), three in Norway (Aalmen et al. 2020; Mathisen et al. 2023, 2024) and one each in Canada (Tse et al. 2022), France (Lévêque et al. 2022), Türkiye (Öcalan et al. 2024), and New Zealand (Clark et al. 2023). Eight studies reported using semi‐structured interviews (Reid et al. 2009; Channa et al. 2019; Räisänen and Hunt 2014; Kinnaird et al. 2019; Ashby and Ogden 2024; Ritholz et al. 2022; Masheb et al. 2024; Banasiak et al. 2007; Aalmen et al. 2020; Mathisen et al. 2023, 2024; Clark et al. 2023), one informal interview (Henderson et al. 2003), two surveys or questionnaires (Durand 2004; Kwok et al. 2023), and four the use of focus groups (DeBate and Tedesco 2006; Herman et al. 2014; Tse et al. 2022; Lévêque et al. 2022) as a method of data collection. One case study (Öcalan et al. 2024) was unclear about its method of data collection. The rest (*N* = 4) (Johnston et al. 2007; Malson et al. 2022; Linville et al. 2010; Wade et al. 2022) used a combination of two or more data collection methods (see Table 1).

**TABLE 1:** Overview of the characteristics of included studies.

Population	References	Location	Participants	Condition	Data collection	Data analysis
HCPs	Henderson et al. (2003)	UK	GPs (*N* = 18)	BED	Informal interviews (45 min)	Constant comparison
	Tse et al. (2022)	Canada	Family medicine residents (*N* = 6), Practicing family physicians (*N* = 5)	EDs	Semi‐structured focus groups (2 × 90 min)	Thematic analysis
	Johnston et al. (2007)	UK	GPs (*N* = 7), Midwives (*N* = 2), Health Visitor (*N* = 1)	EDs	Cross‐sectional face‐to‐face surveys, semi‐structured interviews	Framework approach
	Aalmen et al. (2020)	Norway	GPs (*N* = 5)	EDs	Semi‐structured interviews (40–60 min)	Systematic text condensation
	DeBate and Tedesco (2006)	USA	General dentists (*N* = 21)	EDs	Focus groups (3×)	Qualitative analysis—no further elaboration, discusses themes, and uses modified coding methods developed by Spradley
	Malson et al. (2022)	UK	GPs (*N* = 41) Patients were excluded from the review	EDs	Combination of semi‐structured interviews, focus group discussions, and qualitative surveys	Thematic analysis
	Reid et al. (2009)	UK	GPs (*N* = 20)	EDs	Semi‐structured interviews	Thematic analysis
	Linville et al. (2010)	USA	HCPs in: Family practice (*N* = 8) Obstetrics/gynecology (*N* = 2) Pediatrics (*N* = 2)	EDs	Paper and pencil survey and interviews	Thematic analysis
	Lévêque et al. (2022)	France	GPs (*N* = 23)	EDs	Focus groups (2×)	Grounded theory
	Wade et al. (2022)	Australia	GPs (*N* = 73)	EDs	Semi‐structured telephone interviews and a focus group	Thematic analysis
	Kwok et al. (2023)	Australia	General practitioner/primary care physician (*N* = 5) Endocrinologist (*N* = 2) Pediatrician (*N* = 5) Adolescent physician (*N* = 1) Surgeon (*N* = 6) Other specialist (respiratory, sleep, obesity) (*N* = 1) Nurse (*N* = 3) Dietitian (*N* = 29) (Clinical) psychologist (*N* = 4) Exercise physiologist (*N* = 1) Other (psychotherapist, obesity/feeding research scientist) (*N* = 2)	EDs	Survey (free text responses)	Thematic analysis
	Ashby and Ogden (2024)	UK	GPs (*N* = 14)	EDs	Remote in‐depth semi‐structured interviews	Thematic analysis
HCPs + lived experience	Mathisen et al. (2024)	Norway	MSc exercise physiologists (*N* = 5) Physiotherapists (*N* = 4) Dietitian (*N* = 1) Patients (*N* = 10)	BED (*N* = 6) BN (*N* = 4) (both full threshold)	Semi‐structured interviews	Reflexive thematic analyses
	Durand (2004)	UK	GPs (*N* = 29, *N* = 25) Patient (*N* = 54)	BN (full threshold)	Postal survey (at 3 and 9 months) Questionnaire	Content analysis
Lived experience	Mathisen et al. (2023)	Norway	Patient (*N* = 8)	BED/BN (both full‐threshold)	Semi‐structured interviews	Reflexive thematic analyses
	Masheb et al. (2024)	USA	Patient (*N* = 16)	EDs (self‐reported diagnosis, not further specified)	Phone interviews (30–45 min)	Rapid data analysis
	Öcalan et al. (2024)	Türkiye	Patient (*N* = 1)	BN (full threshold)	Unclear	Unclear
	Patterson‐Norrie et al. (2023)	Australia	Patient (*N* = 12)	Lived experience of EDs (AN + BN = 8, BED = 2, ARFID = 1, OSFED = 1)	Semi‐structured interviews	Thematic analysis
	Channa et al. (2019)	UK	Patient (*N* = 1)	BN (full threshold)	Semi‐structured interview	Interpretative phenomenological analysis
	Banasiak et al. (2007)	Australia	Patient (*N* = 36)	BN (90.7% meeting full DSM‐IV criteria, rest were subthreshold)	Semi‐structured interviews	Content analysis
	Clark et al. (2023)	New Zealand	Patient (*N* = 13), Whānau members (*N* = 2)	Lived experience of EDs (A*N* = 8, BN = 4, BED = 1)	Semi‐structured interviews (In line with Kaupapa Māori research, interviews followed the hui process)	Thematic analysis
	Herman et al. (2014)	USA	Patient (*N* = 25)	BED (full threshold)	Focus groups (3× diagnosed, 3× undiagnosed, but meeting DSM‐5 criteria)	Qualitative analysis—no further elaboration, use of Atlas.ti software
	Räisänen and Hunt (2014)	UK	Patient (*N* = 10)	EDs, a mixture of undiagnosed, self‐identified, and full threshold (AN = 4, AN and EDNOS = 2, BN = 3, BN and AN = 1)	Semi‐structured interviews	Qualitative interpretative approach (combination of inductive close reading and constant comparison)
	Kinnaird et al. (2019)	UK	Patient (*N* = 14)	EDs, full‐threshold (AN = 7, BN = 4, BED = 2, EDNOS = 1)	Semi‐structured interviews (15–30 min)	Thematic analysis
	Ritholz et al. (2022)	USA	Patient (*N* = 21)	BED + Type 2 diabetes (full‐threshold)	Semi‐structured interviews	Thematic analysis

*Note*: NB: If not specified otherwise, no further clarification was reported about the type of thematic analysis used or the threshold level of the condition of the live experience sample.

Most studies (*N* = 13) used thematic analysis (Malson et al. 2022; Reid et al. 2009; Kinnaird et al. 2019; Ashby and Ogden 2024; Linville et al. 2010; Ritholz et al. 2022; Wade et al. 2022; Patterson‐Norrie et al. 2023; Kwok et al. 2023; Mathisen et al. 2023, 2024; Tse et al. 2022; Clark et al. 2023) as the method of analysis, while the rest (*N* = 7) used grounded theory (Lévêque et al. 2022), constant comparison (Henderson et al. 2003), a framework approach (Johnston et al. 2007), systematic text condensation (Aalmen et al. 2020), interpretative phenomenological analysis (Channa et al. 2019), content analysis (Durand 2004; Banasiak et al. 2007), rapid data analysis (Masheb et al. 2024) and an interpretative approach combining inductive close reading and constant comparison (Räisänen and Hunt 2014). Three studies (DeBate and Tedesco 2006; Herman et al. 2014; Öcalan et al. 2024) did not report on their specific methods apart from stating that they did qualitative analysis.

Fourteen articles reported on HCP perspectives, and 13 on those from patients. Eight studies sampled GPs (Henderson et al. 2003; Malson et al. 2022; Reid et al. 2009; Ashby and Ogden 2024; Durand 2004; Wade et al. 2022; Aalmen et al. 2020; Lévêque et al. 2022), four a mix of HCPs (Johnston et al. 2007; Linville et al. 2010; Kwok et al. 2023; Tse et al. 2022), one therapist (Mathisen et al. 2024) and one general dentist (DeBate and Tedesco 2006). Out of these 14 studies, 11 focused on EDs in general (Johnston et al. 2007; Malson et al. 2022; Reid et al. 2009; Ashby and Ogden 2024; DeBate and Tedesco 2006; Linville et al. 2010; Wade et al. 2022; Kwok et al. 2023; Aalmen et al. 2020; Tse et al. 2022; Lévêque et al. 2022), 1 on BED/BN (Mathisen et al. 2024), 1 on BN (Durand 2004), and 1 on BED (Henderson et al. 2003). In the case of patients' perspectives, eight studies sampled women exclusively (Channa et al. 2019; Durand 2004; Ritholz et al. 2022; Banasiak et al. 2007; Patterson‐Norrie et al. 2023; Mathisen et al. 2023, 2024; Öcalan et al. 2024), two men exclusively (Räisänen and Hunt 2014; Kinnaird et al. 2019), and three a mix of genders, with a primarily female‐dominated sample (Herman et al. 2014; Masheb et al. 2024; Clark et al. 2023). Overall, the pooled sample consisted of 336 HCPs and 223 individuals with lived experience. Few studies reported on the socioeconomic status or demographic characteristics of participants. For more details about the participant characteristics (see Table 2).

**TABLE 2:** Overview of the sample characteristics of included studies.

Population	References	Age	Gender	SES	Race	Ethnicity	Other
HCPs	Henderson et al. (2003)	NR	F = 9, M = 9	NR	NR	NR	NR
	Tse et al. (2022)	NR	F = 8, M = 3	NR	NR	NR	Year of residency, Years of independent practice
	Johnston et al. (2007)	NR	NR	NR	NR	NR	NR
	Aalmen et al. (2020)	NR	F = 1, M = 4	NR	NR	NR	Years of experience as a GP
	DeBate and Tedesco (2006)	NR	F = 4, M = 17	NR	NR	NR	Years of practice
	Malson et al. (2022)	NR	F = 37, M = 4	NR	NR	NR	Type of GPs
	Reid et al. (2009)	NR	F = 10, M = 10	NR	NR	NR	Place of work
	Linville et al. (2010)	NR	F = 8, M = 4	NR	Caucasian	NR	Type of HCP
	Lévêque et al. (2022)	Age groups 25–35 (15) 35–45 (5) > 45 (3)	F = 10, M = 13	NR	NR	NR	Mode of exercise, Department of exercise
	Wade et al. (2022)	NR	NR	NR	NR	NR	NR
	Kwok et al. (2023)	18–25 years (*N* = 3) 26–34 years (*N* = 6) 35–49 years (*N* = 29) 50–64 years (*N* = 18) 65 + years (*N* = 3)	F = 45, M = 13, Prefer not to say = 1	NR	NR	NR	Primary Place of Employment, SEIFA IRSAD of primary practice location, Treatment age group, Aspects of consultations and different treatment approaches
	Ashby and Ogden (2024)	NR	F = 14	NR	White	British	Years of qualification, geographical area of work, GP role
HCPs + lived experience	Mathisen et al. (2024)	HCP: NR LE: Mean = 32.9 (SD = 5.2)	HCP: F = 9, M = 1 LE: F = 10	HCP: NR LE: NR	NR	HCP + LE: Nordic ethnicity	HCP: NR LE: EDE‐Q global score, self‐reported duration of illness
	Durand (2004)	HCP: NR LE: NR^a^	HCP: NR LE: F = 54	HCP: NR LE: NR^a^	HCP: NR LE: NR^a^	NR	NR^a^
Lived experience	Mathisen et al. (2023)	NR^a^	NR^a^	NR^a^	NR^a^	NR^a^	NR^a^
	Masheb et al. (2024)	Mean = 53.8 Range: 25–73	F = 8, M = 8	NR	NR	NR	Region, Mental Health Diagnosis, Self‐reported ED diagnosis
	Öcalan et al. (2024)	24	F = 1	Attended university, unclear of current status	NR	NR	Description of family relationships, education history
	Patterson‐Norrie et al. (2023)	Mean = 36.9	F = 12	Employment status Employed, full‐time (*N* = 3) Employed, part‐time (*N* = 5) Unemployed (*N* = 4) Highest level of education Master's degree (*N* = 1) with other degrees not quantified	NR	NR	Years of ED experience, State of residence
	Channa et al. (2019)	Early 20s	F = 1	Contact with ED services in an urban area	South Asian Indian	NR	Family history and upbringing
	Banasiak et al. (2007)	NR^a^	F = 36	NR^a^	NR^a^	NR	NR^a^
	Clark et al. (2023)	16–65	F = 14, M = 1	NR	NR	NR	NR
	Herman et al. (2014)	Mean = 42.92 (SD = 10.61) Range: 24–72	F = 15, M = 10	Employment status Employed, full‐time (*N* = 13) Employed, part‐time (*N* = 6) Homemaker (*N* = 1) Student (*N* = 1) Retired (*N* = 1) Disabled (*N* = 3) Highest level of education Secondary/high school (*N* = 6) Associate degree (*N* = 5) College degree (*N* = 7) Graduate degree (*N* = 4) Other (*N* = 3) Annual household income < $25,000 (*N* = 3) $26,000–$34,000 (*N* = 4) $35,000–$50,000 (*N* = 6) $51,000–$75,000 (*N* = 5) $76,000–$99,000 (*N* = 4) > $100,000 (*N* = 3)	White (*N* = 12), Black/African American (*N* = 10), Hispanic (*N* = 3)	NR	Physical comorbidities, psychiatric comorbidities
	Räisänen and Hunt (2014)	17–25	M = 10	Student (*N* = 6), Employed (*N* = 2), Unemployed (*N* = 2)	White British (*N* = 8), Latino (*N* = 1), Mixed (*N* = 1)	NR	Type of ED, Age at diagnosis, sexuality
	Kinnaird et al. (2019)	Mean = 29.43 years (SD = 8.55)	M = 14	NR	NR	NR	Type of ED, length of ED, comorbidities, treatment status, type of treatment received
	Ritholz et al. (2022)	Mean = 49 (19–66)	F = 21	NR	90% non‐Hispanic white	NR	Mean body mass index, mean HbA1c, Mean diabetes duration

Abbreviation: NR, not reported.

^a^ Characteristics not available for the included sample.

### 3.3 Risk of Bias/Quality

Four studies were considered of good quality with a low risk of bias, while the rest (*N* = 21) were considered to be of moderate quality with a medium risk of bias. The most common issue was a lack of adequate consideration of reflexivity in the included studies (see Supplementary File 3).

### 3.4 Thematic Synthesis

Thematic synthesis identified four main themes that describe the factors impacting the identification and management of BED and BN in primary care. These main themes were: Knowledge and understanding of BED and BN (Galmiche et al. 2019), Effective Communication in HCP–patient Interactions (Hay et al. 2023), Attitudes and emotions (Giel et al. 2022), and Treatment and referral pathways in practice (Milano et al. 2018): gaps, disconnect, and clarity. All four themes were present in both HCP and patient‐focused articles. The data provided below are quotes from the participants who had taken part in the primary studies, which were used by the authors of the included studies to illustrate their findings.

#### 3.4.1 Knowledge and Understanding of BED and BN

Twenty papers from both patient and HCP perspectives considered knowledge and understanding of diagnostic criteria, symptoms, presentation of BED and BN, comorbidities, and treatment options to influence the identification and management of BED and BN in primary care. HCPs reported limited knowledge of the diagnostic criteria for different EDs.

I thought that was similar to Bulimia. In fact, I haven't made any distinction in my mind, so my understanding was that binge eaters also vomit, make themselves vomit, and that those are the bulimics. (Henderson et al. 2003)

Henderson et al. (2003) and Mathisen et al. (2024) highlight that this lack of knowledge is attributed to a lack of training but also to limited exposure to patients with BED and BN. Moreover, identification of BED and BN was identified to be more challenging where comorbidities exist, such as obesity, diabetes, or other psychiatric illnesses (Henderson et al. 2003; Reid et al. 2009; Wade et al. 2022; Kwok et al. 2023; Tse et al. 2022; Lévêque et al. 2022). For example, Tse et al. (2022) described cases of patients where, due to prior obesity and bariatric surgery, disordered eating was difficult to identify. A GP in Lévêque et al. (2022) called this scenario a “chicken‐and‐egg question” in which HCPs struggle to identify the primary underlying condition, in this case, BED and BN, and establish a causal relationship with the comorbidities. A lack of identification of the symptoms of BED and BN by HCPs was seen to delay treatment. The problem of comorbidities was also supported by accounts from the patient's perspective (Herman et al. 2014; Ritholz et al. 2022; Clark et al. 2023); for example, Clark et al. (2023) describe a case of a patient with bipolar disorder who was unable to get help for their ED until symptoms of bipolar disorder had been managed.

Patients were aware of the lack of knowledge and understanding of BED and BN among HCPs and reported experiencing negative interactions with primary care because of this lack of knowledge (Herman et al. 2014; Ritholz et al. 2022; Masheb et al. 2024; Banasiak et al. 2007; Patterson‐Norrie et al. 2023; Mathisen et al. 2024; Clark et al. 2023). HCPs' limited knowledge of BED and BN is evidenced by reports of GPs using their “gut feeling” to diagnose (Clark et al. 2023) or giving inappropriate advice, such as to “push food away” (Herman et al. 2014) or to lose weight (Masheb et al. 2024) while disregarding the interaction between diabetes and BED (Ritholz et al. 2022). These forms of advice were seen to negatively impact patients' well‐being and the identification and management of their EDs. A few studies also reported on patients' limited understanding of the symptoms (Herman et al. 2014; Masheb et al. 2024; Patterson‐Norrie et al. 2023; Mathisen et al. 2024) and management (Öcalan et al. 2024) of BED and BN, especially among men (Räisänen and Hunt 2014) and people of color (Channa et al. 2019). For example, a male patient in Räisänen and Hunt (2014) is quoted to say:

I didn't know men could get eating disorders then …… I didn't know the symptoms, didn't know anything, it was just, to me it was just happening.

Where good knowledge and understanding of BED and BN existed, individuals were able to be identified early and get timely treatment, resulting in enhanced recovery and improved engagement in treatment (Durand 2004; Masheb et al. 2024; Patterson‐Norrie et al. 2023; Öcalan et al. 2024). Patients in Ritholz et al. (2022) suggested that treatment engagement could be improved by increasing HCPs' understanding of the complexity of BED and BN comorbidities, such as diabetes.

#### 3.4.2 Effective Communication in HCP–Patient Interactions

Twenty papers discussed HCP–patient interactions, including HCPs' communication skills about BED/BN and patients' feelings of being heard. HCPs reported a lack of confidence (Johnston et al. 2007; Malson et al. 2022; Reid et al. 2009; DeBate and Tedesco 2006; Linville et al. 2010; Wade et al. 2022; Tse et al. 2022; Lévêque et al. 2022) to address BED/BN when talking with patients presenting in primary care, resulting in discomfort (Johnston et al. 2007; DeBate and Tedesco 2006; Tse et al. 2022) and anxiety (Tse et al. 2022) among HCPs. HCPs attributed this lack of confidence to limited ED‐specific training (Johnston et al. 2007; Reid et al. 2009; Wade et al. 2022; Tse et al. 2022; Lévêque et al. 2022) and limited exposure to BED/BN cases (Ashby and Ogden 2024). For example, Linville et al. (2010) reported that HCPs struggled with identifying what questions to ask their patients in relation to BED/BN, while DeBate and Tedesco (2006) and Lévêque et al. (2022) highlighted that HCPs found the mere act of asking BN/BED questions difficult and were not sure what approaches were appropriate to touch on/introduce BED/BN into their conversations with patients. However, Wade et al. (2022) reported that some GPs found screening tools helpful in establishing such conversations with patients. While screening tools were recognized as being potentially helpful for some HCPs (Johnston et al. 2007; Malson et al. 2022; Linville et al. 2010; Wade et al. 2022; Aalmen et al. 2020) and patients (Masheb et al. 2024), others were uncertain about using BED/BN screening tools in their practice (Masheb et al. 2024; Lévêque et al. 2022), with no further elaboration offered. Durand (2004) reported that GPs found using a self‐help manual for BN treatment helpful in improving their understanding of BN and their confidence to address BN.

The anxiety experienced by HCPs in relation to bringing up BED/BN was attributed to concerns about overdiagnosing or offending patients, while potentially jeopardizing the patient–HCP relationship (DeBate and Tedesco 2006; Wade et al. 2022; Tse et al. 2022).

I worry about bringing something up and then labelling somebody and then them not wanting to talk about it. So, I think maybe subconsciously I am avoiding it at times, which is terrible—right? Because we want to pick it up so, that's what I struggle with. (Tse et al. 2022)

A HCP in Lévêque et al. (2022) used the analogy of “opening Pandora's box,” in which HCPs were wary of the potential harm they could cause by talking about BED and BN, such as triggering an unhealthy relationship with eating or issues HCPs cannot or do not want to deal with. On the other hand, in one paper, GPs reported that having good continuity of care can mitigate potential communication issues and enhance the patient–HCP relationship (Ashby and Ogden 2024).

Patients noticed the lack of confidence and potential dismissiveness in HCPs' communication about BED/BN, which was attributed to a lack of knowledge and understanding of BED/BN. For example, some patients described their interaction with HCPs as “robotic” (Ritholz et al. 2022) and *“*not therapeutic” (Öcalan et al. 2024) when discussing BED/BN. Hence, patients may experience shame and embarrassment when trying to address their binge eating symptoms (Herman et al. 2014; Masheb et al. 2024; Patterson‐Norrie et al. 2023; Mathisen et al. 2024). If HCPs do not have sufficient communication skills and confidence to discuss BED/BN, patients may not feel heard and feel dismissed instead, potentially impacting help‐seeking behavior and access to care, as evidenced by Clark et al. (2023):

I remember quite plainly one GP (general practitioner) saying to me ‘look don't worry, I don't think there's anything going on, I usually get a feeling up the back of my neck when it's something like an eating disorder and it's not that’.

Thus, despite HCP concerns, “opening Pandora's box” is needed to ensure patients feel they are able to discuss their ED symptoms in a safe space without feeling judged (avoiding shame and embarrassment) (Masheb et al. 2024; Patterson‐Norrie et al. 2023).

#### 3.4.3 Attitudes and Emotions

Twenty‐one articles reported a mixture of attitudes on different topics related to the identification and management of BED and BN (e.g., family, beliefs about BED/BN, and treatment and referral pathways) from both the patient and HCP perspectives.

The studies reported evidence of various beliefs about BED/BN among HCPs, such as BED/BN being akin to alcohol addiction (Reid et al. 2009; Lévêque et al. 2022) or BED/BN being a “phase” (Linville et al. 2010; Lévêque et al. 2022), possibly due to a lack of knowledge and understanding. These beliefs and attitudes were seen to negatively impact the quantity and quality of support offered, as described by the authors (Henderson et al. 2003; Räisänen and Hunt 2014; Herman et al. 2014; Clark et al. 2023). Patient accounts showed the impact that HCP attitudes had on their experience of care (Räisänen and Hunt 2014; Herman et al. 2014; Banasiak et al. 2007; Clark et al. 2023). For example, individuals with a higher weight (including obesity) (Herman et al. 2014; Masheb et al. 2024; Banasiak et al. 2007; Clark et al. 2023), who were older (Banasiak et al. 2007), were of non‐western culture (Clark et al. 2023), or were male (Räisänen and Hunt 2014; Clark et al. 2023), were not seen to be considered by HCPs for a BED/BN diagnosis. However, one patient reported a positive HCP attitude, during which “he [the HCP] fought for me a lot with my diagnosis” (Masheb et al. 2024).

Moreover, as reported by the authors, HCP beliefs about the importance of early treatment and positive outcomes of treatment on the individual could impact HCP approaches to identification using screening (Johnston et al. 2007; Malson et al. 2022; Reid et al. 2009; Linville et al. 2010; Wade et al. 2022; Tse et al. 2022; Lévêque et al. 2022). A HCP in Johnstone et al. (Johnston et al. 2007) is quoted to say: “I don't know whether there is any advantage in picking them up early.” Patients were reported as being aware of the influence of negative false beliefs and the passive approach to screening (Räisänen and Hunt 2014; Clark et al. 2023).

Some studies did highlight that some HCPs reflected on how their own beliefs impacted their practice. For example, Tse et al. (2022) and Wade et al. (2022) reported the acknowledgement of the impact of weight bias on BED/BN identification, while Johnston et al. (2007) reported the impact of culture on the presentation of symptoms and beliefs of individuals. Several studies reported a willingness among HCPs to undergo training to improve knowledge and confidence in addressing and managing BED/BN (Ashby and Ogden 2024; Durand 2004; Linville et al. 2010; Aalmen et al. 2020; Mathisen et al. 2024). Such a reflective and empathetic approach to care delivery was encouraged by patients who reported that appropriate acknowledgement of BED/BN experiences by HCPs can create a safe and judgment‐free environment, enabling patients to receive and engage with care (Durand 2004; Banasiak et al. 2007).

The authors reported that family is perceived by patients and HCPs as both a negative and positive factor in the facilitation of attitudes in relation to the identification and management of BED/BN. Family members were seen as able to support individuals with BED/BN during help‐seeking and treatment, facilitating an open discussion between HCPs and the patient, and a positive attitude (Reid et al. 2009; Channa et al. 2019; Ashby and Ogden 2024; DeBate and Tedesco 2006; Aalmen et al. 2020; Clark et al. 2023). On the other hand, family members were also recognized as being able to facilitate negative attitudes by acting as a barrier to help‐seeking and the development of a relationship between HCPs and patients (Malson et al. 2022; Channa et al. 2019; DeBate and Tedesco 2006; Linville et al. 2010; Aalmen et al. 2020; Clark et al. 2023).

HCPs reported frustration about patients' secretive behavior and beliefs, such as the implications of accepting psychiatric care, as it was seen to delay care delivery (Henderson et al. 2003; Johnston et al. 2007; Malson et al. 2022; Reid et al. 2009; Aalmen et al. 2020; Tse et al. 2022; Lévêque et al. 2022). Studies of patient perspectives reported disagreement with this view (Channa et al. 2019; Räisänen and Hunt 2014; Herman et al. 2014; Ritholz et al. 2022; Öcalan et al. 2024; Clark et al. 2023) and explained that secretive behavior can be a mechanism to protect patients from judgment (Räisänen and Hunt 2014; Patterson‐Norrie et al. 2023), especially from the HCP (Durand 2004; Herman et al. 2014; Ritholz et al. 2022; Masheb et al. 2024; Patterson‐Norrie et al. 2023), as described by a female patient with BED in Ritholz et al. (2022):

I felt ashamed, and I would avoid reporting more than I had to. So sometimes I would wait until the next appointment to tell them. And I felt ashamed that I was doing this to myself.

However, some HCPs acknowledged that this secretive behavior was a symptom of BED/BN (Malson et al. 2022; Reid et al. 2009; Aalmen et al. 2020) and was likely to be fueled by existing stigma around BED/BN (Channa et al. 2019; Räisänen and Hunt 2014; Herman et al. 2014; Ritholz et al. 2022; Tse et al. 2022; Clark et al. 2023).

Studies reported on HCPs' frustration with the referral process (Johnston et al. 2007; Ashby and Ogden 2024; Tse et al. 2022), resulting in long waitlists and patient deterioration. As reported by the authors, this situation left HCPs to deal with complex cases beyond their scope (Henderson et al. 2003; Johnston et al. 2007; Malson et al. 2022; Reid et al. 2009; Ashby and Ogden 2024; Linville et al. 2010; Aalmen et al. 2020; Tse et al. 2022) and discouraged them from screening for BED/BN (Linville et al. 2010). For example, Tse et al. (2022) described a case in which the patient was not “sick enough” to be referred for ED services but was also too sick to be left untreated.

We've been in a case recently with a patient in my practice where they were too sick for outpatient, they weren't sick enough for inpatient …… those patients fall through the cracks‐ who sees them, who takes care of them? It's us, but we haven't had the training to be able to do it. (Tse et al. 2022)

The frustration with the limited access to ED services was shared by patients (Masheb et al. 2024). A patient in Clark et al. (2023) described how BED/BN was seen as not worthy of treatment due to AN being at the “top of the hierarchy” due to visible physical signs and potential severity.

#### 3.4.4 Treatment and Referral Pathways in Practice: Gaps, Disconnects, and Clarity

Twenty‐three papers emphasized the crucial impact the existence, accessibility, and clarity of treatment and referral pathways had on the identification and management of BED and BN in primary care.

HCPs reported limited knowledge about existing treatment and referral pathways. For example, in DeBate and Tedesco (2006), dentists reported having dysfunctional communication about BED/BN referrals with GPs:

My experience has been in the past that we've referred a patient, a young girl, to her physician and the physician told the mother, ‘I wish these dentists would stop diagnosing Bulimia with all the patients’. The patient left the office; they were frustrated with us because we had recommended. Maybe there are some concerns that maybe you should see a physician, and they [the physician] just dismissed it that it wasn't a problem.

A lack of evidence‐based, clear guidance on management and referral was seen to discourage HCPs from screening and identifying BED/BN, leading to inaccessibility of treatment for patients (Johnston et al. 2007; Linville et al. 2010; Wade et al. 2022). Hence, HCPs emphasized the need for transparent and concrete guidance on actions when positively screening patients with BED/BN (Johnston et al. 2007; Ashby and Ogden 2024; DeBate and Tedesco 2006; Wade et al. 2022; Tse et al. 2022).

Although some awareness was seen to exist around treatment and referral pathways, their accessibility for all HCPs and patients was limited due to a lack of resources, such as time (Malson et al. 2022; Durand 2004; DeBate and Tedesco 2006; Linville et al. 2010; Herman et al. 2014; Wade et al. 2022; Banasiak et al. 2007; Aalmen et al. 2020; Clark et al. 2023), finances (Masheb et al. 2024; Patterson‐Norrie et al. 2023) and specialized support (Johnston et al. 2007; Reid et al. 2009; Durand 2004; Tse et al. 2022; Lévêque et al. 2022), referral criteria (Ashby and Ogden 2024; Kwok et al. 2023), and dysfunctional connections between primary care and secondary care (Malson et al. 2022; Ashby and Ogden 2024; Durand 2004; Linville et al. 2010; Kwok et al. 2023; Aalmen et al. 2020). HCPs in Linville et al. (2010) and Aalmen et al. (2020) explained that consultation time was not enough to introduce new topics, such as BED/BN, leading to a focus on prioritizing physical health issues, with BED/BN also sometimes being the lowest priority within mental health. Patients reported awareness of not being a priority because of their BED/BN or limited time available for HCPs and distress due to inaccessibility to treatment (Durand 2004; Herman et al. 2014; Banasiak et al. 2007; Clark et al. 2023). To address time issues, dentists suggested involving dental hygienists in the identification process (Mathisen et al. 2023), while other HCPs suggested restructuring time frames and staffing in GP practices (Aalmen et al. 2020).

Both patients and HCPs reported limited access to ED services and a lack of specialized staff to support treatment (Henderson et al. 2003; Johnston et al. 2007; Malson et al. 2022; Reid et al. 2009; Räisänen and Hunt 2014; Kinnaird et al. 2019; Ashby and Ogden 2024; Linville et al. 2010; Herman et al. 2014; Masheb et al. 2024; Banasiak et al. 2007; Patterson‐Norrie et al. 2023; Aalmen et al. 2020; Tse et al. 2022; Lévêque et al. 2022; Clark et al. 2023).

Her [GP] struggle was that they haven't got the services to give to you. They want to but they just haven't got them. (Räisänen and Hunt 2014)

Räisänen and Hunt (2014) explained that the inaccessibility to ED services caused long waiting lists for treatment, leading to frustration for both patients and HCPs, as described in the previous theme, and resulting in patient disengagement with treatment (Ashby and Ogden 2024; Clark et al. 2023). Further accessibility issues were reported by both patients and some HCPs, such as problems with the type of treatment offered, including method or length (Kinnaird et al. 2019; Ashby and Ogden 2024; Durand 2004; Herman et al. 2014; Masheb et al. 2024; Banasiak et al. 2007; Kwok et al. 2023; Clark et al. 2023) and the place of treatment, such as in rural or urban areas (Patterson‐Norrie et al. 2023; Clark et al. 2023). Patients in Räisänen and Hunt (2014) and Kinnaird et al. (2019) reported a lack of inclusivity for men in treatment options and activities and highlighted that a female‐dominated environment, including staff and peers in support groups, can lead to self‐consciousness in some male patients and disengagement with treatment.

I would like [treatment materials] to address gender in slightly different ways. Instead of having a chapter on gender and body image, I mean I understand all that and I think it's important, but I would simply like to have in the rest of the material examples that take into account men of all sorts. (Kinnaird et al. 2019)

To offset the limited access to ED services, some patients suggested using primary care‐based management options, such as a community‐focused group intervention (Mathisen et al. 2023, 2024) or self‐help treatment (Durand 2004). However, some GPs raised concerns about primary care‐based self‐help treatment as it is time‐consuming for GPs and poses as a barrier for patients to access in‐depth counseling (Durand 2004). As reported by the authors, access to suitable treatment can motivate patients to seek help and engage with treatment (Durand 2004; Masheb et al. 2024; Mathisen et al. 2023, 2024; Öcalan et al. 2024).

A disconnect between primary and secondary care was reported by both HCPs and patients. HCPs described a lack of or too much communication from ED services (Malson et al. 2022; Linville et al. 2010; Aalmen et al. 2020). A patient in Clark et al. (2023) emphasized the impact of this disconnection on patients:

More flow between eating disorders and other services, more connection because from my point of view I feel that eating disorders is quite separate and it's hard to get them involved and waitlist times are horrible. If I'm struggling and I get referred back there it's like oh cool it's going to be a three month wait and I'm like well, by the time it gets to that three months I'm like nah—whatever.

On the contrary, good connections between primary and secondary care, as reported by Aalmen et al. (2020), created a smooth continuity in patients' care and made follow‐ups easier for HCPs. This kind of connection in the care pathway was championed by both HCPs (Linville et al. 2010; Aalmen et al. 2020; Mathisen et al. 2024) and patients (Ritholz et al. 2022; Clark et al. 2023).

Clarity about HCPs' roles and responsibilities in BED/BN treatment and referral pathways was observed to be lacking, causing confusion about expectations between both patients and HCPs (Henderson et al. 2003; Malson et al. 2022; Reid et al. 2009; Ashby and Ogden 2024; Durand 2004; Lévêque et al. 2022). Interestingly, only dentists in one study reported clarity in their roles and responsibilities in the identification and management of BED/BN (DeBate and Tedesco 2006). While some HCPs reported BED/BN management being out of their remit (Henderson et al. 2003), others reported a desire to have a clear definition of their roles and responsibilities and to receive specific guidance on BED/BN management in primary care to aid limited knowledge and training (Malson et al. 2022; Ashby and Ogden 2024; Linville et al. 2010; Tse et al. 2022; Lévêque et al. 2022). Tse et al. (2022) reported that clear roles and responsibilities improved continuity of care and follow‐ups.

What is your role as the family doctor? You are identifying it; you are doing the physical part and the electrolyte part. But then there's also the psychotherapy part and that is the huge part of the treatment. That's probably the most challenging part and I think I feel unequipped. (Tse et al. 2022)

Patients provided more specific descriptions of the expectations of HCPs' roles and responsibilities in the management of BED/BN but not in their identification (Räisänen and Hunt 2014; Kinnaird et al. 2019; Banasiak et al. 2007). Patients emphasized that access to different gendered HCPs and positive involvement of HCPs in BED/BN management, including providing a judgment‐free environment, reassurance, motivation, empathy, and continuity of care, enhanced engagement with treatment and increased the possibility of recovery (Räisänen and Hunt 2014; Kinnaird et al. 2019; Ritholz et al. 2022; Banasiak et al. 2007; Clark et al. 2023). On the contrary, Banasiak et al. (2007) reported that minimal HCP involvement in the implementation of a treatment program and limited skills to manage psychological issues reduced patient compliance with treatment and motivation.

Articles from the patients' perspective offered other themes, such as perspectives on gender or suggestions on improvement of a specific guided self‐help tool, that were not included in this synthesis as they were not relevant to the question of this review.

### 3.5 Linking the Themes: The Interacting Factors Influencing the Identification and Management of BED and BN in Primary Care

**FIGURE 3:**
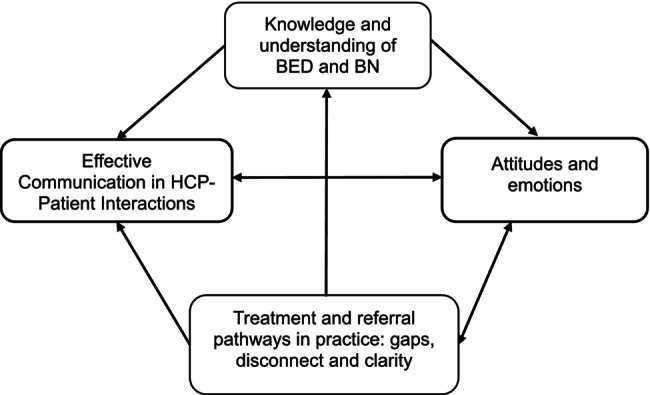
The model of factors impacting the identification and management of BED and BN.

We developed a conceptual model of identification and management of BED/BN (Figure 3) that linked the themes from the thematic synthesis. In this model, the identified factors are closely interlinked, suggesting that there is no single factor that would improve or create a barrier to BED/BN identification and management when considered in isolation.

## 4 Discussion

This systematic review aimed to explore the perceptions of HCPs and patients in relation to the identification and management of BED and BN in primary care. Our synthesis highlights multiple factors and interacting influences at both individual and system levels.

This review found that there is limited knowledge of BED/BN among HCPs and some patients as well. The literature on mental health literacy reports low ED‐specific literacy among both HCPs (Johansson et al. 2015; Worsfold and Sheffield 2018; Seah et al. 2018, 2017) and the public (Gratwick‐Sarll et al. 2013; Bullivant et al. 2020; O'Connor et al. 2016), which corresponds with our findings. The literature, however, focuses on EDs overall and, apart from a few small studies (Hollett and Carter 2021; Supina et al. 2016; Mond et al. 2008), fails to address BED and BN specifically. Hence, a systematic review calls for expanding mental health literacy on BED and BN (Bullivant et al. 2020) as compared to other mental health conditions, such as depression, given both HCPs (McNicholas et al. 2016) and the public (Mond et al. 2008, 2010) have poorer knowledge and more negative attitudes toward BED and BN.

In this review, gut feeling diagnosis seemed to be linked to limited knowledge and negative attitudes toward BED/BN. Conversely, in wider primary care literature, gut feeling appears to play a positive and important role in the diagnosis of some disorders, such as cancer (Yao et al. 2023; Smith et al. 2020; Stolper et al. 2011). In cases where GPs have longstanding relationships with their patients, and continuity of care is maintained, gut feelings may be more informed and therefore more accurate, particularly if subtle behavioral or physical changes are noticed over time (Alyafei and Al Marri 2020). However, the scope for gut feeling may be limited in the context of EDs, where patients often have infrequent contact with primary care or experience poor continuity, as suggested in our findings. In such circumstances, gut feeling is less likely to be a reliable or trusted tool. Moreover, there is limited understanding of gut feeling diagnosis in the context of mental health, and it remains a negative experience reported by patients. Hence, no further conclusions can be made about gut feeling diagnosis for this context, and further research is needed.

Our findings unraveled differences in perceptions about secretive behavior. While HCPs viewed it as a barrier to care delivery, patients perceived it as a protective mechanism against judgment from HCPs. While the literature does report secretive eating as a behavior associated with BED and BN (Lydecker et al. 2019; Goss and Allan 2009; Kass et al. 2017), no studies address the mechanisms of secretiveness and its role for patients. Hence, further research is needed to understand the function of secretiveness for patients with BED and BN and to identify potential recommendations for the clinical practice of HCPs.

Our findings revealed that the limited existence, accessibility, and clarity of treatment and referral pathways can hinder HCPs' knowledge of BED/BN and HCP–patient interactions, and cause frustration to both patients and HCPs. Supporting our findings, Winston et al. (2007) reported that the presence of ED services in close proximity to GP surgeries can improve knowledge and understanding of EDs in HCPs. However, they also highlighted that GPs seem to have more knowledge of ED management due to being forced to deal with the cases for which no evidence was identified in our review. However, recently there has been a push to close the gap between primary and secondary care services to address the management of complex mental health cases (Dunn et al. 2024), including BED and BN, and foster more integrated care approaches (Mitchell et al. 2015, 2020; Rugkåsa et al. 2020) which focus on close collaboration of primary care staff with others, such as ED services (Johns et al. 2019) or psychologists (Rugkåsa et al. 2020), to reduce the burden on GPs. These recent efforts in health services could potentially explain the contradictory findings. However, further research is needed to explore the current situation in primary care.

Our findings identified the main factors influencing the identification and management of BED and BN (limited knowledge and understanding, attitudes, HCP–patient interactions, and limited treatment and referral pathways). The impact of these factors on identification and management is supported by wider literature (Parker et al. 2020), such as on ADHD (Kamimura‐Nishimura et al. 2023; French et al. 2020; Baweja et al. 2021). This means that the barriers are not unique to BED/BN, and perhaps more general measures on a wider level need to be taken to address them. Improvements are being made in specific areas, such as mapping ADHD services or improving identification and management of ADHD using new technology (Price et al. 2020; Peterson et al. 2024; De Silva et al. 2021). These improvements highlight potential ways to tackle issues reported in this review for BED and BN and contribute to more widespread change in health services for mental health conditions. A good example of how barriers and patients' interactions with health services have been addressed is in the field of depression. The establishment of clear treatment and referral pathways, increased provision of services through initiatives such as NHS Talking Therapies in the UK, and the introduction of depression into the UK Quality and Outcomes Framework (Lester and Campbell 2010) led to improved knowledge about depression, better communication skills, and attitudes over time among primary care staff and the public (Curran et al. 2023; Mitchell et al. 2009). Overall, there is evidence from wider mental health literature that the barriers reported in this review can be successfully tackled at a general health service level, such as incentivization, and at a more disorder‐specific level, such as the use of digital health to tackle gaps in treatment pathways.

### 4.1 Strengths and Limitations

The main strengths of this study are (a) the systematic identification and synthesis of qualitative literature, (b) the integration of HCPs' and patients' perspectives into one narrative, and (c) the involvement of PPI and interest holders in data analysis and interpretation. The main limitation is the lack of papers that focused on BED or BN or specified their results separately for BED or BN when focusing on all EDs. As a result, these findings were derived from studies that included patients with BED and BN in their sample or as part of discussions with HCPs but did not analyze them separately. Future studies should report on EDs separately to provide primary data on BED/BN specifically. There were also a few papers discussing the experiences of HCPs other than GPs/family physicians. Hence, the findings of this review primarily reflect the experiences of GPs and family physicians. While it is acknowledged that GPs are the primary HCPs identifying and managing BED/BN, it is important to highlight this limitation as initial identification and referral to GPs/family physicians can be made by other primary care professionals, whose experience is just as crucial to consider when viewing primary care holistically. Finally, the variable quality of included studies may have influenced the findings, highlighting the need for better‐reported and more rigorous research.

### 4.2 Implications for Practice and Future Directions

Based on the findings of this review, a summary of recommendations is presented in Table 3.

**TABLE 3:** Summary of recommendations based on the synthesized evidence.

Area for recommendation	Topic	Recommendation
Clinical practice	Training and Education	Develop and offer comprehensive training programs and support for current and incoming HCPs to (Galmiche et al. 2019) improve knowledge of BED and BN diagnostic criteria, symptoms, treatment options and comorbidities associated with BED and BN, such as obesity, diabetes, and psychiatric conditions; and (Hay et al. 2023) develop effective communication strategies for addressing BED and BN with patients sensitively and without stigmatization, including the potential use of screening tools to instigate initial conversations.
		Evaluate the training to examine the quality of care provided and clinical outcomes.
	Integrated Care and Collaboration	Promote better collaboration among HCPs in primary care to ensure a continuous referral and care pathway, including considering the role of all primary care professionals in the identification of BED and BN.
		Promote better communication between primary care HCPs and ED specialists to ensure better continuity of care, reduce pressure on GPs by considering the use of primary care mental health services, provide support via digital health, and improve awareness of BED/BN.
	Clarity about Treatment and Referral Pathways	Establish and disseminate clear guidelines (existing or new) and protocols for the referral and treatment of patients with BED and BN for HCPs.
		Ensure all HCPs are aware of and have easy access to these pathways to provide timely and appropriate care.
Research		Distinguish findings for BED and BN from AN and other EDs to ensure relevance of future research to BED and BN. This would enable the production of more specific and better‐quality evidence for BN, and especially BED, both of which have been under‐researched, with direct implications for clinical outcomes.
		Explore current practices around identifying and managing BED and BN in primary care and address the gaps in specific guidelines and care pathways for HCPs in relation to BED and BN.
		Address the lack of reporting and inclusivity by including people of all ages, genders, cultures, and weights that were highlighted in this review in future research to improve the applicability and scalability of findings.
Public	Education	Raise awareness about BED and BN (signs, symptoms, health consequences, and available treatment options) to improve public understanding and transparency of care pathways and encourage early help‐seeking behavior. Provide culturally sensitive and gender‐specific resources to cater to diverse patient needs and to address gaps highlighted in this review.

## 5 Conclusion

In conclusion, this is the first synthesis of qualitative work that explored the perspectives of HCPs and patients on identifying and managing BED and BN in primary care. This review provides an overview of the main factors and their interactions impacting the identification and management of BED and BN in primary care, including knowledge and understanding, HCP–patient interactions, attitudes and emotions, and treatment and referral pathways. Overall, a coordinated strategy is required that includes comprehensive and evaluated training for HCPs, improved interdisciplinary collaboration and continuity of care, the establishment of clear referral and treatment pathways, targeted and inclusive research distinguishing between BED and BN, and public education initiatives to raise awareness and reduce stigma.

## Author Contributions

**Stella Kozmér:** conceptualization, methodology, formal analysis, writing – review and editing, writing – original draft, project administration, investigation. **Christopher O'Rouke:** writing – review and editing, investigation. **Natalia S. Lawrence:** conceptualization, methodology, writing – review and editing, supervision, project administration. **Jane R. Smith:** supervision, project administration, writing – review and editing, conceptualization, methodology. **Samantha B. van Beurden:** supervision, project administration, writing – review and editing, writing – original draft, methodology, conceptualization, formal analysis, visualization.

## Conflicts of Interest

The authors declare no conflicts of interest.

## Supporting information


**Data S1:** Supporting Information.

## Data Availability

The review used data previously published (publicly available) only. No primary data were collected for this publication. Further information can be obtained from the corresponding author(s).
