# Supplementary material for: Identification and Management of Binge‐Eating Disorder and Bulimia Nervosa in Primary Care Settings: A Qualitative Systematic Review of Healthcare Professionals' and Patients' Perceptions
Source: Int J Eat Disord. 2025 Oct 14;59(1):16–34. doi: 10.1002/eat.24568 (PMC12773673; doi:10.1002/eat.24568)
Supplement: Supplementary file 1 — Data S1: Supporting Information. [file EAT-59-16-s001.docx]

**Supplementary File 1.**

**Table 4**. PRISMA checklist

| **Section and Topic** | | | **Item #** | | **Checklist item** | | **Location where item is reported** | |
| --- | --- | --- | --- | --- | --- | --- | --- | --- |
| **TITLE** | | | | |  | |  |  |
| Title | 1 | | Identify the report as a systematic review. | | 1 | |  |  |
| **ABSTRACT** | | | | |  | |  |  |
| Abstract | 2 | | See the PRISMA 2020 for Abstracts checklist. | | 4 | |  |  |
| **INTRODUCTION** | | | | |  | |  |  |
| Rationale | 3 | | Describe the rationale for the review in the context of existing knowledge. | | 5-7 | |  |  |
| Objectives | 4 | | Provide an explicit statement of the objective(s) or question(s) the review addresses. | | 7 | |  |  |
| **METHODS** | | | | |  | |  |  |
| Eligibility criteria | 5 | | Specify the inclusion and exclusion criteria for the review and how studies were grouped for the syntheses. | | 7 | |  |  |
| Information sources | 6 | | Specify all databases, registers, websites, organisations, reference lists and other sources searched or consulted to identify studies. Specify the date when each source was last searched or consulted. | | 8 | |  |  |
| Search strategy | 7 | | Present the full search strategies for all databases, registers and websites, including any filters and limits used. | | 8 | |  |  |
| Selection process | 8 | | Specify the methods used to decide whether a study met the inclusion criteria of the review, including how many reviewers screened each record and each report retrieved, whether they worked independently, and if applicable, details of automation tools used in the process. | | 8 | |  |  |
| Data collection process | 9 | | Specify the methods used to collect data from reports, including how many reviewers collected data from each report, whether they worked independently, any processes for obtaining or confirming data from study investigators, and if applicable, details of automation tools used in the process. | | 9 | |  |  |
| Data items | 10a | | List and define all outcomes for which data were sought. Specify whether all results that were compatible with each outcome domain in each study were sought (e.g. for all measures, time points, analyses), and if not, the methods used to decide which results to collect. | | 9 | |  |  |
|  | 10b | | List and define all other variables for which data were sought (e.g. participant and intervention characteristics, funding sources). Describe any assumptions made about any missing or unclear information. | | 9 | |  |  |
| Study risk of bias assessment | 11 | | Specify the methods used to assess risk of bias in the included studies, including details of the tool(s) used, how many reviewers assessed each study and whether they worked independently, and if applicable, details of automation tools used in the process. | | 9 | |  |  |
| Effect measures | 12 | | Specify for each outcome the effect measure(s) (e.g. risk ratio, mean difference) used in the synthesis or presentation of results. | | NA | |  |  |
| Synthesis methods | 13a | | Describe the processes used to decide which studies were eligible for each synthesis (e.g. tabulating the study intervention characteristics and comparing against the planned groups for each synthesis (item #5)). | | 9 | |  |  |
|  | 13b | | Describe any methods required to prepare the data for presentation or synthesis, such as handling of missing summary statistics, or data conversions. | | NA | |  |  |
|  | 13c | | Describe any methods used to tabulate or visually display results of individual studies and syntheses. | | 9 | |  |  |
|  | 13d | | Describe any methods used to synthesize results and provide a rationale for the choice(s). If meta-analysis was performed, describe the model(s), method(s) to identify the presence and extent of statistical heterogeneity, and software package(s) used. | | 9-10 | |  |  |
|  | 13e | | Describe any methods used to explore possible causes of heterogeneity among study results (e.g. subgroup analysis, meta-regression). | | NA | |  |  |
|  | 13f | | Describe any sensitivity analyses conducted to assess robustness of the synthesized results. | | NA | |  |  |
| Reporting bias assessment | 14 | | Describe any methods used to assess risk of bias due to missing results in a synthesis (arising from reporting biases). | | 9 | |  |  |
| Certainty assessment | 15 | | Describe any methods used to assess certainty (or confidence) in the body of evidence for an outcome. | | 9 | |  |  |
| **RESULTS** | | | | |  | |  |  |
| Study selection | 16a | | Describe the results of the search and selection process, from the number of records identified in the search to the number of studies included in the review, ideally using a flow diagram. | | 11 | |  |  |
|  | 16b | | Cite studies that might appear to meet the inclusion criteria, but which were excluded, and explain why they were excluded. | | 12 | |  |  |
| Study characteristics | 17 | | Cite each included study and present its characteristics. | | 13-20 | |  |  |
| Risk of bias in studies | 18 | | Present assessments of risk of bias for each included study. | | 21 | |  |  |
| Results of individual studies | 19 | | For all outcomes, present, for each study: (a) summary statistics for each group (where appropriate) and (b) an effect estimate and its precision (e.g. confidence/credible interval), ideally using structured tables or plots. | | NA | |  |  |
| Results of syntheses | 20a | | For each synthesis, briefly summarise the characteristics and risk of bias among contributing studies. | | 21-32 | |  |  |
|  | 20b | | Present results of all statistical syntheses conducted. If meta-analysis was done, present for each the summary estimate and its precision (e.g. confidence/credible interval) and measures of statistical heterogeneity. If comparing groups, describe the direction of the effect. | | NA | |  |  |
|  | 20c | | Present results of all investigations of possible causes of heterogeneity among study results. | | NA | |  |  |
|  | 20d | | Present results of all sensitivity analyses conducted to assess the robustness of the synthesized results. | | NA | |  |  |
| Reporting biases | 21 | | Present assessments of risk of bias due to missing results (arising from reporting biases) for each synthesis assessed. | |  | |  |  |
| Certainty of evidence | 22 | | Present assessments of certainty (or confidence) in the body of evidence for each outcome assessed. | | NA | |  |  |
| **DISCUSSION** | | | | |  | |  |  |
| Discussion | 23a | | Provide a general interpretation of the results in the context of other evidence. | | 32-35 | |  |  |
|  | 23b | | Discuss any limitations of the evidence included in the review. | | 32-35 | |  |  |
|  | 23c | | Discuss any limitations of the review processes used. | | 35 | |  |  |
|  | 23d | | Discuss implications of the results for practice, policy, and future research. | | 35-37 | |  |  |
| **OTHER INFORMATION** | | | | |  | |  |  |
| Registration and protocol | 24a | | Provide registration information for the review, including register name and registration number, or state that the review was not registered. | | 7 | |  |  |
|  | 24b | | Indicate where the review protocol can be accessed, or state that a protocol was not prepared. | | 7 | |  |  |
|  | 24c | | Describe and explain any amendments to information provided at registration or in the protocol. | | NA | |  |  |
| Support | 25 | | Describe sources of financial or non-financial support for the review, and the role of the funders or sponsors in the review. | | 2 | |  |  |
| Competing interests | 26 | | Declare any competing interests of review authors. | | 2 | |  |  |
| Availability of data, code and other materials | 27 | | Report which of the following are publicly available and where they can be found: template data collection forms; data extracted from included studies; data used for all analyses; analytic code; any other materials used in the review. | | 2 | |  |  |

**Supplementary File 2.**

**List 1**. Full search strategy (NB: Mesh terms were adjusted according to the relevant database)

| 1. | Bulimia/ or Binge-Eating Disorder/ or Bulimia Nervosa/ or "Feeding and Eating Disorders"/ |
| --- | --- |
| 2. | BED.ti,ab,kw. |
| 3. | bing*.ti,ab,kw. |
| 4. | bulimi*.ti,ab,kw. |
| 5. | purg*.ti,ab,kw. |
| 6. | BN.ti,ab,kw. |
| 7. | (eating adj3 disorder*).ti,ab,kw. |
| 8. | (appetite adj3 disorder*).ti,ab,kw. |
| 9. | (feeding adj3 disorder*).ti,ab,kw. |
| 10. | General Practitioners/ |
| 11. | general practi*.ti,ab,kw. |
| 12. | family practi*.ti,ab,kw. |
| 13. | GP*.ti,ab,kw. |
| 14. | (primary adj2 care).ti,ab,kw. |
| 15. | primary healthcare.ti,ab,kw. |
| 16. | psychological wellbeing practitioner*.ti,ab,kw. |
| 17. | mental health practitioner*.ti,ab,kw. |
| 18. | PWP*.ti,ab,kw. |
| 19. | IAPT.ti,ab,kw. |
| 20. | community pharmacist*.ti,ab,kw. |
| 21. | nurse practitioner*.ti,ab,kw. |
| 22. | practice nurse*.ti,ab,kw. |
| 23. | family physician*.ti,ab,kw. |
| 24. | view*.ti,ab,kw. |
| 25. | experience*.ti,ab,kw. |
| 26. | knowledge.ti,ab,kw. |
| 27. | attitude*.ti,ab,kw. |
| 28. | opinion*.ti,ab,kw. |
| 29. | perception*.ti,ab,kw. |
| 30. | belief*.ti,ab,kw. |
| 31. | thought*.ti,ab,kw. |
| 32. | perspective*.ti,ab,kw. |
| 33. | qualitative.ti,ab,kw. |
| 34. | focus group*.ti,ab,kw. |
| 35. | ethnograph*.ti,ab,kw. |
| 36. | 1 or 2 or 3 or 4 or 5 or 6 or 7 or 8 or 9 |
| 37. | 10 or 11 or 12 or 13 or 14 or 15 or 16 or 17 or 18 or 19 or 20 or 21 or 22 or 23 |
| 38.  39. | 24 or 25 or 26 or 27 or 28 or 29 or 30 or 31 or 32 or 33 or 34 or 35  36 and 37 and 38 |

**Supplementary File 3.**

**Table 5**. Quality assessment of included studies

| Article | Clear aims | Appropriate methodology | Appropriate research design | Appropriate recruitment strategy | Data collection addressed the research issues | Adequate consideration of reflexivity | Ethical issues | Sufficient rigour of data analysis | Clear statement of findings | Valuable research | Total |
| --- | --- | --- | --- | --- | --- | --- | --- | --- | --- | --- | --- |
| Henderson et al.(33) | Yes | Yes | 3 | 2 | 3 | 1 | 2 | 3 | 3 | 3 | 20 |
| Tse et al.(54) | Yes | Yes | 3 | 3 | 3 | 3 | 3 | 3 | 3 | 3 | 24 |
| Johnston et al.(34) | Yes | Yes | 3 | 3 | 3 | 3 | 3 | 3 | 3 | 3 | 24 |
| Aalmen et al.(51) | Yes | Yes | 1 | 3 | 3 | 2 | 3 | 2 | 3 | 3 | 20 |
| DeBate & Tedesco (42) | Yes | Yes | 3 | 3 | 3 | 1 | 3 | 1 | 2 | 3 | 19 |
| Malson et al.(35) | Yes | Yes | 3 | 3 | 3 | 1 | 2 | 2 | 3 | 3 | 20 |
| Reid et al.(36) | Yes | Yes | 3 | 2 | 3 | 1 | 3 | 3 | 3 | 3 | 21 |
| Linville et al.(43) | Yes | Yes | 3 | 3 | 3 | 1 | 3 | 3 | 3 | 3 | 22 |
| Lévêque et al.(55) | Yes | Yes | 2 | 3 | 3 | 1 | 2 | 2 | 3 | 3 | 19 |
| Wade et al.(47) | Yes | Yes | 3 | 3 | 3 | 1 | 3 | 2 | 3 | 3 | 21 |
| Kwok et al.(50) | Yes | Yes | 3 | 3 | 3 | 1 | 3 | 2 | 3 | 3 | 21 |
| Ashby & Ogden(40) | Yes | Yes | 3 | 2 | 3 | 3 | 3 | 3 | 3 | 3 | 23 |
| Mathisen et al.(52) | Yes | Yes | 3 | 3 | 3 | 1 | 3 | 3 | 3 | 3 | 22 |
| Durand (41) | Yes | Yes | 3 | 3 | 3 | 1 | 3 | 3 | 3 | 3 | 22 |
| Mathisen et al.(53) | Yes | Yes | 3 | 3 | 3 | 1 | 3 | 3 | 3 | 3 | 22 |
| Channa et al.(37) | Yes | Yes | 3 | 3 | 3 | 2 | 3 | 3 | 3 | 3 | 23 |
| Banasiak et al.(48) | Yes | Yes | 3 | 3 | 3 | 3 | 3 | 2 | 2 | 3 | 22 |
| Clark et al. (57) | Yes | Yes | 3 | 3 | 3 | 3 | 3 | 3 | 3 | 3 | 24 |
| Herman et al.(44) | Yes | Yes | 3 | 3 | 3 | 2 | 3 | 3 | 3 | 3 | 23 |
| Räisänen & Hunt(38) | Yes | Yes | 2 | 3 | 3 | 1 | 3 | 3 | 3 | 3 | 21 |
| Kinnaird et al.(39) | Yes | Yes | 2 | 3 | 3 | 1 | 3 | 3 | 3 | 3 | 21 |
| Ritholz et al.(45) | Yes | Yes | 3 | 3 | 3 | 2 | 3 | 3 | 3 | 3 | 23 |
| Masheb et al. (46) | Yes | Yes | 3 | 3 | 3 | 2 | 3 | 3 | 3 | 3 | 23 |
| Öcalan et al.(56) | Yes | Yes | 3 | 3 | 3 | 3 | 3 | 1 | 3 | 2 | 21 |
| Patterson-Norrie et al.(49) | Yes | Yes | 3 | 3 | 3 | 3 | 3 | 3 | 3 | 3 | 24 |

NB: Quality categorised as total score >15 – weak, 16-23 – moderate, 24 – strong.

**Supplementary File 4.** List of excluded studies

| **Reference** | **Exclusion Reason** |
| --- | --- |
| Al-Sulti (2022). Assessment of Knowledge, Attitude, and Practices (KAP) Towards Eating Disorders Among Sultan Qaboos University Students | No qualitative data |
| Anstine & Grineko (2000). Rapid screening for disordered eating in college-aged females in the primary care setting | No qualitative data |
| Ballou (2008). Polarity therapy as a complementary treatment for bulimia nervosa | Not based in primary care |
| Banas et al. (2013). Eating disorder training and attitudes among primary care residents | No qualitative data |
| Banas et al. (2009). Eating disorder training and attitudes in primary care residents | No qualitative data |
| Barnes et al. (2014). An examination of weight bias among treatment-seeking obese patients with and without binge eating disorder. | No qualitative data |
| Barnett et al. (2016). Using group therapy to support eating disordered mothers with their children: The relevance for primary care | No information on identification or management |
| Bergeson (2025). Improving nurse practitioner students' knowledge about eating disorders | No qualitative data |
| Beveridge et al. (2015). Back to ED basics-GP and health practitioner eating disorders education program | Oral presentation |
| Boone (2014). Awakened to a life: an existential-phenomenological examination of the lived experience of recovery from eating disorders | No information on identification or management |
| Boule & McSherry (2002). Patients with eating disorders. How well are family physicians managing them? | No qualitative data |
| Braaten (2011). Eating disorders | Book chapter |
| Breland et al. (2016). Women veterans' treatment preferences for disordered eating | No information specific to BED or BN |
| Brennan (2018). Cognitive behavioural intervention for obesity | Book chapter |
| Broussard (2002). The experience of bulimia: A phenomenologic investigation | No information on identification or management |
| Bryant et al. (2024). Identifying eating disorders at the earliest opportunity: Testing the reliability of an Online Eating Disorder Screener (IOI-S) in primary care and youth mental health settings | No qualitative data; patients were under 16 at the time of identification and/or management |
| Bursten et al. (1996). Detecting and treating bulimia nervosa: how involved are family physicians? | No qualitative data |
| Byrom et al. (2022). Seeking support for an eating disorder: A qualitative analysis of the university student experience-accessibility of support for students | No differentiation between eating disorders and the majority of participants had AN |
| Chen et al. (2003). Comparison of group and individual cognitive-behavioral therapy for patients with bulimia nervosa | No qualitative data |
| Chew & Temples (2022). Adolescent Eating Disorders: Early Identification and Management in Primary Care | Review |
| Chung & Sangvai (2021). Primary care-based treatment for eating disorders | Book chapter |
| Clarke & Pollimeni-Walker (2004). Treating Individuals with Eating Disorders in Family Practice: A Needs Assessment | No qualitative data |
| Clark et al. (2023).  'E koekoe te Tui, e ketekete te Kaka, e kuku te Kereru, The Tui chatters, the Kaka cackles, and the Kereru coos': Insights into explanatory factors, treatment experiences and recovery for Maori with eating disorders - A qualitative study | No eating disorder characteristics recorded and therefore no information specific to BED or BN |
| Coales et al. (2024). Can guided self-help improve the management of binge eating in adults type 2 diabetes? Results of the POSE-D study | Conference abstract |
| Crow et al. (2004). A Survey of Binge Eating and Obesity Treatment Practices among Primary Care Providers | No qualitative data |
| Cruse (2005). Physician awareness of eating disorders from the patient's perspective | No qualitative data |
| Currin (2006). Primary care treatment of eating disorders: from diagnosis to referral | No qualitative data |
| Currin et al. (2009). Primary care physicians' knowledge of and attitudes toward the eating disorders: do they affect clinical actions? | No qualitative data |
| Davis (2010). Patients' perspectives on eating disorder treatment: A resource for practitioners | No qualitative data; No information specific to BED or BN |
| de Carvalho et al. (2016). Therapeutic itineraries of individuals with symptoms of anorexia and bulimia | Not based in primary care |
| DeSocio et al. (2007). Screening for childhood eating disorders in primary care | Patients were under 16 at the time of identification and/or management |
| Devantier (2018). Exploring the processes of change in individual cognitive behavioural therapy for bulimia nervosa from the patients' perspectives: a grounded theory study | Patients were under 16 at the time of identification and/or management |
| Diamond Allen (2024). Increasing primary care providers' knowledge, confidence, and screening for eating disorders | Patients were under 16 at the time of identification and/or management; no qualitative data |
| Dichter et al. (2002). Bulimia nervosa: knowledge, awareness, and skill levels among advanced practice nurses | No qualitative data |
| Di Pietro et al. (2005). Eating Disorders in Primary Care Service: Survey on cases at risk for Anorexia and Bulimia Nervosa in collaboration with General Practitioner of Naples suburban area | No qualitative data |
| Doherty & McNamee (2015). General practitioner knowledge, skills and attitudes to eating disorders | Poster presentation |
| Eisenberg & Neumark-Sztainer (2010). Friends' Dieting and Disordered Eating Behaviors Among Adolescents Five Years Later: Findings From Project EAT | No qualitative data |
| Fang (2021). Improving Binge Eating Disorder Screening in Primary Care Settings | No qualitative data |
| Faux (2025). Implementing Screening for Eating Disorders of College Age Male Athletes in a University's Student Health Center | Conference abstract |
| Forbush et al. (2023). The Building Healthy Eating and Self-Esteem Together for University Students Mobile App to Treat Eating Disorders: User-Centered Research Design and Feasibility Study | No qualitative data; Not based in primary care |
| Foye and Bartel (2021). Consider male eating disorders: A create approach to improving treatment in primary health care for men and boys with eating disorders | Conference abstract |
| Franklin (2020). A clinical training on eating disorders for community healthcare providers and stakeholders. | No qualitative data |
| Gilsbach et al. (2024). Lost in between-the transition process from a child and adolescent eating disorder service to adult mental health services in the German health care system | Study is focused on AN |
| Goldfield (1998). A comparison of eating attitudes and behavior and general psychological characteristics in bulimics and bodybuilders | No qualitative data |
| Goldfield et al. (2006). Body image, binge eating, and bulimia nervosa in male bodybuilders | No qualitative data |
| Goldstein-Kerzner (1996). Examination of components of cognitive-behavior therapy in the treatment of bulimia nervosa | No qualitative data |
| Gooding et al. (2017). Implementation and evaluation of two educational strategies to improve screening for eating disorders in pediatric primary care | No qualitative data |
| Gordon & Sefler (2024). 50. Barriers to Screening and Diagnosis of Eating Disorders in the Pediatric Primary Care Setting | Conference abstract |
| Greenfield (1995). The effects of dentists' knowledge, skills, and attitudes/beliefs regarding bulimia nervosa on their recognition, intervention, and referral practices: An exploratory study | No qualitative data |
| Green et al. (2008). General practitioner attitudes towards referral of eating-disordered patients: a vignette study based on the theory of planned behaviour | No qualitative data |
| Grogan et al. (2021). A qualitative study on the multi-level process of resilience development for adults recovering from eating disorders | No eating disorder characteristics recorded; healthcare providers were not based in primary care |
| Gurney & Halmi (2001). An eating disorder curriculum for primary care providers | No qualitative data |
| Gurney et al. (2000). Eating disorders | Review |
| Halvorsen et al. (2014). Patients' experience of their general practitioner's follow-up of serious eating disorders | No qualitative data |
| Hawkins (1996). The relationship between self-concept and eating disorder symptomatology in adolescent schoolgirls | Patients were under 16 at the time of identification and/or management |
| Hay et al. (2007). Knowledge and beliefs about bulimia nervosa and its treatment: A comparative study of three disciplines | No qualitative data |
| Hay et al. (2005). Bulimia nervosa mental health literacy of general practitioners. | No qualitative data |
| Herman et al. (2017). Use and Value of the 7-Item Binge Eating Disorder Screener in Clinical Practice | No qualitative information specific to primary care |
| Heywood-Everett (2006). General practitioners' diagnosis, management and referral of patients with an eating disorder | No qualitative data; no information specific to BED or BN |
| Huang (2015). Motivational patterns and related psychopathology in Chinese patients with eating disorders | Patients were under 16 at the time of identification and/or management |
| Hurst et al. (2017). Bulimia nervosa in adolescents: a new therapeutic frontier | No primary care professionals |
| Johns et al. (2019). Current eating disorder healthcare services-The perspectives and experiences of individuals with eating disorders, their families and health professionals: Systematic review and thematic synthesis. | Systematic review |
| Johnson et al. (2013). A Subject and Witness Perspective of the Effectiveness of Current Treatment Practices for Eating Disorders | Not based in primary care |
| Johnson (1993). Psychodynamic treatment of anorexia nervosa and bulimia. | Book chapter |
| Jones et al. (2012). Who benefits most from guided self-help for binge eating? An investigation into the clinical features of completers and non-completers | No qualitative data |
| Kaitz et al. (2020). Barriers in addressing body image and eating issues in primary care: An overview of women's narratives | No eating disorder characteristics recorded and therefore no information specific to BED or BN |
| Kass et al. (2023). Clinician perspectives on assessing for disordered eating in adolescents and young adults with cystic fibrosis | No information specific to primary care |
| Kempley (1988). Effectiveness of an exposure treatment for bulimia | No qualitative data |
| King et al. (2023). Qualitative exploration of factors influencing women veterans' disordered eating symptoms and treatment preferences in VHA primary care | BED/BN cannot be identified; focus on disordered eating behaviour |
| Koziarska-Rościszewska et al. (2021). Eating disorders in university students in lodz, the role of a family physician | No qualitative data |
| Kreipe & Mou (2000). Eating disorders in adolescents and young adults | Review |
| Lafrance Robinson et al. (2013). "i want help!": Psychologists' and physicians' competence, barriers, and needs in the management of eating disorders in children and adolescents in Canada | No information specific to BED or BN; Patients were under 16 at the time of identification and/or management |
| Lange (2016). Eating disorder screening, evaluation, and referral in the primary care setting | No information specific to BED or BN |
| Lazare et al. (2021). Exploring the primary care experiences of adult patients with eating disorders: A qualitative analysis | No eating disorder characteristics recorded and therefore no information specific to BED or BN |
| Lebow, Narr, et al. (2021). Engaging primary care providers in managing pediatric eating disorders: A mixed methods study | Patients were under 16 at the time of identification and/or management |
| Lebow, O’Brien et al. (2021). A primary care modification of family-based treatment for adolescent restrictive eating disorders | Patients were under 16 at the time of identification and/or management; no information specific to BED or BN |
| Linville, Ayoama, & Gau (2012). The effectiveness of a brief eating disorder training programme in medical settings | No qualitative data |
| Linville, Brown, & O’Neil (2012). Medical providers' self perceived knowledge and skills for working with eating disorders: A national survey | No qualitative data |
| Lubarda et al. (2017). Binge eating disorder management: Can medical education improve physician knowledge? | No qualitative data |
| Maguen et al. (2025). Provider and leader perspectives on eating disorder screening and the importance of a clinical pathway in the Veterans Health Administration: A qualitative study | No information specific to primary care |
| Mandell et al. (2022). “Exploring the Relationships of Psychological Characteristics and Disordered Eating Behaviors with Dieting Among College Students” | No qualitative data |
| Manti et al. (2025). Adult eating disorders integrated mental health service (aedimhs): An evaluation of a new primary care intervention model | No eating disorder characteristics recorded and therefore no information specific to BED or BN |
| McCarvill & Weaver (2014). Primary care of female adolescents with type 1 diabetes mellitus and disordered eating | Discussion paper |
| McClure (2020). Screening for identification of binge eating disorder in adolescents | Patients were under 16 at the time of identification and/or management |
| McNicholas et al. (2016). Stigma and treatment of eating disorders in Ireland: Healthcare professionals' knowledge and attitudes | No qualitative data |
| Meadows et al. (1986). Eating attitudes and disorder in young women: A general practice based survey | No qualitative data |
| Mitrofan et al. (2019). Care experiences of young people with eating disorders and their parents: Qualitative study | Primarily AN focus; no information specific to BED or BN |
| Mond et al. (2007). Mental health literacy and eating-disordered behavior: Beliefs of adolescent girls concerning the treatment of and treatment-seeking for bulimia nervosa | Patients were under 16 at the time of identification and/or management |
| Mond & Hay (2008). Public perceptions of binge eating and its treatment | No qualitative data |
| Mond, Hay et al. (2008). Mental health literacy and eating disorders: What do women with bulimic eating disorders think and know about bulimia nervosa and its treatment? | No qualitative data |
| Mond, Myers et al. (2008). 'Excessive exercise' and eating-disordered behaviour in young adult women: Further evidence from a primary care sample | No qualitative data |
| Montano et al. (2016). Diagnosing binge eating disorder in a primary care setting | Review |
| Monteleone et al. (2024). Attitudes and gaps in knowledge of the diagnosis, treatment, and psychopathology of eating disorders among different health professionals | No qualitative data |
| Morris and Harrison (2008). What can a GP do? Management of eating disorders in primary care | Book chapter |
| Mühleck et al. (2020). Online survey on the awareness of offers for information, prevention, counselling, and aftercare for eating disorders | No ED characteristics reported |
| Muscari (1998). Thin line: managing care for adolescents with anorexia and bulimia. | No primary data |
| Muscari (1996). Primary care of adolescents with bulimia nervosa | Review |
| Noble (2023). Screening for eating disorders in pediatric primary care | Patients were under 16 at the time of identification and/or management |
| Nowaskie et al. (2021). Eating disorder symptomatology in transgender patients: Differences across gender identity and gender affirmation | No qualitative data |
| Omondi et al. (2025). Exploring mirror perspectives among African American women living with obesity at a primary care clinic in the United States | No information specific to BED or BN |
| Peat & Feltner (2022). Addressing eating disorders in primary care: Understanding screening recommendations and opportunities to improve care | Forum |
| Pietrabissa et al. (2014). A Brief Strategic Therapy protocol for Binge Eating Disorder | Study protocol |
| Radunz et al. (2024). Addressing the gap of early intervention for eating disorders in primary health care | No qualitative data |
| Raffoul et al. (2022). Evaluation of a one-hour asynchronous video training for eating disorder screening and referral in U.S. pediatric primary care: A pilot study | No qualitative data |
| Richmond et al. (2020). How do you define recovery? A qualitative study of patients with eating disorders, their parents, and clinicians | Patients were under 16 at the time of identification and/or management |
| Rommel et al. (2013). Emotional differentiation and parental bonding in inpatients suffering from eating disorders | No qualitative data |
| Rose & Waller (2017). Cognitive-behavioral therapy for eating disorders in primary care settings: Does it work, and does a greater dose make it more effective? | No qualitative data |
| Sangha et al. (2019). Eating disorders in males: how primary care providers can improve recognition, diagnosis, and treatment | Review |
| Scionti (2001). An investigation into individuals with eating disorders and their perceptions of family functioning, depression, and seasonal affective disorder in an outpatient population | No qualitative data |
| Scrandis & Arnow (2023). Binge-eating disorder | Review |
| Shaikh & Kayani (2014). Detection of eating disorders in 16-20 year old female students - perspective from Islamabad, Pakistan | No qualitative data |
| Shortland et al. (2018). Seeking help in general practice for people with eating disorders | Conference abstract |
| Silverstein et al. (2019). Impact of an oral health education intervention among a group of patients with eating disorders (anorexia nervosa and bulimia nervosa) | No qualitative data; Patients were under 16 at the time of identification and/or management |
| Sitarz (2021). Understanding the experiences of young adults receiving community-based eating disorder care | HCPs were not based in primary care |
| Smith & Harrop (2024). "That's not at all what I needed" trans adults' perspectives on trans-affirming primary care and eating disorders | No information specific to BED or BN |
| Steiger et al. (1987). Perspectives on the prevention of anorexia nervosa and bulimia | Review |
| Supina et al. (2016). Knowledge of binge eating disorder: a cross-sectional survey of physicians in the United States | No qualitative data |
| Tan & Spector-Hill (2021). Identifying and managing eating disorders in type 1 diabetes mellitus: An all-Wales study | No qualitative data; no information specific to BED or BN |
| Thaler et al. (2018). A tertiary-care/primary-care partnership aimed at improving care for people with eating disorders | No qualitative data |
| Tiereney et al. (2009). Caring for people with type 1 diabetes mellitus engaging in disturbed eating or weight control: A qualitative study of practitioners' attitudes and practices | HCPs were not based in primary care |
| Todd (1992). Development and validation of a systematic training program for the diagnosis of anorexia nervosa, bulimia nervosa, and concomitant conditions | No qualitative data; HCPs were not based in primary care |
| Tramontana (2008). The connection between spirituality and recovery from bulimia nervosa | No information on identification or management |
| Troop et al. (1994). WAYS OF COPING IN WOMEN WITH EATING DISORDERS | No qualitative data; no information specific to primary care |
| Vega Alonso et al. (2001). Eating disorders: A descriptive study in Primary Care | No qualitative data |
| Verbeek and Schmid (2019). Challenges in Outpatient Psychotherapeutic Care for Eating Disorders in Children and Adolescents: A Clinical Practitioner's View | Forum |
| Vermassen et al. (2012). Eating disorders: Diagnosis and treatment by the general practitioner | Review |
| Webb & Schmidt (2021). Facilitators and barriers to supporting young people with eating disorders during their transition to, and time at, university: An exploration of clinicians' perspectives | HCPs were not based in primary care |
| Weber & Davis (2012). Food for thought: Enabling and constraining factors for effective rural eating disorder service delivery | No qualitative data |
| Wilfley et al. (1998). Adapting interpersonal psychotherapy to a group format (IPT-G) for binge eating disorder: Toward a model for adapting empirically supported treatments | No qualitative data |
| Wilhelm & Clarke (1998). Eating disorders from a primary care perspective | Review |
| Wilkins (2002). A training model for effective diagnosis and treatment of eating disorders by family physicians | No information specific to BED or BN |
| Winston et al. (2007). Effect of a specialist eating disorders service on the knowledge and attitudes of local health professionals | No qualitative data |
| Worsfold & Sheffield (2018). Eating disorder mental health literacy: What do psychologists, naturopaths, and fitness instructors know? | HCPs were not based in primary care |
| Zeeuws et al. (2019). Patient's and general practitioner's perspectives regarding disturbed eating. | No qualitative data |
| Zucker et al. (2006). A group parent-training program: A novel approach for eating disorder management. | No qualitative data |
